# Metastatic Spread of Neuroblastoma to the Left Atrium Mimicking Atrial Myxoma: A Rare Occurrence in an Adolescent

**DOI:** 10.7759/cureus.12799

**Published:** 2021-01-20

**Authors:** Zobia Aijaz, Hafiza M Durrani, Pulwasha Iftikhar, Raja Ram Khenhrani, Mohammed FaisalUddin

**Affiliations:** 1 Internal Medicine, Dow University of Health and Sciences, Karachi, PAK; 2 Pediatrics, Dr. Ruth Pfau Civil Hospital Karachi/Dow University of Health and Sciences, Karachi, PAK; 3 Obstetrics and Gynecology, St. John's University, New York, USA; 4 Internal Medicine, Liaquat University of Medical and Health Sciences, Karachi, PAK; 5 Internal Medicine, Deccan College of Medical Sciences, Hyderabad, IND

**Keywords:** neuroblastoma, cardiac mass, adolescent, echocardiography

## Abstract

Neuroblastoma is the most common childhood malignancy arising from the sympathetic neuroblast cells. The most common sites of origin are the adrenal glands and paravertebral regions. However, the involvement of the heart is a rare occurrence in adolescents. Here, we report a case of a 12-year-old male child who was misdiagnosed as a case of cardiac myxoma on initial presentation. Following surgical resection and histological examination, neuroblastoma was revealed. This case report highlights the differential diagnosis for the cardiac mass in an adolescent with an unknown primary origin and also the importance of tissue histopathology for the diagnosis and management of neuroblastoma.

## Introduction

Neuroblastoma (NB), a tumor originating from neural crest cells that are designated for the development of postganglionic sympathetic neurons, encompasses the most common malignancy among extracranial solid childhood tumors. The total burden of neuroblastoma is one in 8,000 live births, which constitutes 6%-10% among all childhood tumors and causing 12-15% mortalities related to malignancies in childhood [1]. Although this tumor is more prevalent in infantile ages, its incidence decreases after five years of age. It has been reported frequently in all age groups [2-5]. Neuroblastoma in the elderly has a distinct clinical behavior in terms of location of the primary tumor, metastatic spread, and response to treatment. Children older than 10 years have different clinicopathological characteristics and complex DNA microarray, which might explain the complexity involved in its presentation and outcomes accompanied by neuroblastoma in the older aged population [6]. Neuroblastoma is a highly metastasizing tumor, and its spread is usually confined to the lymph nodes, bone marrow, liver, and brain in younger children [7]. By contrast, there are few unknown metastatic sites mentioned in their older counterparts. Here, we encountered a case of cardiac metastasis of neuroblastoma in an adolescent male, which masqueraded as left atrial myxoma based on echocardiographic findings. However, histopathological examination of excised mass was consistent with the diagnosis of neuroblastoma. To the best of our knowledge, this is a unique case as the metastatic spread of neuroblastoma to the heart is underreported. A meticulous search for the pertinent documentation provided only one article, which is a case report, enlightening the spread of neuroblastoma to the right atrium through the inferior vena cava [8].

## Case presentation

A 12-year-old male presented to the Emergency Department with complaints of shortness of breath associated with a persistent cough, weight loss, and intermittent low-grade fever for the past few months. The patient had no significant past or family medical history. On physical examination, the pale-looking adolescent male had a temperature of 38.4 degree Celsius, blood pressure of 111/59 mmHg, heart rate of 117 beats/minute, respiratory rate of 30 breaths/minute, and oxygen saturation (SpO_2_) of 92% while breathing ambient air. The cardiopulmonary examination was unremarkable, with no murmurs or audible extra heart sounds. All other physical examinations and laboratory findings were unremarkable. After initial stabilization, baseline investigations were performed. Laboratory studies were pertinent for normocytic normochromic anemia with hemoglobin of 11 g/dL. Blood culture showed no growth of organisms. All other values within complete blood count and complete metabolic panel were unremarkable.

**Figure 1:**
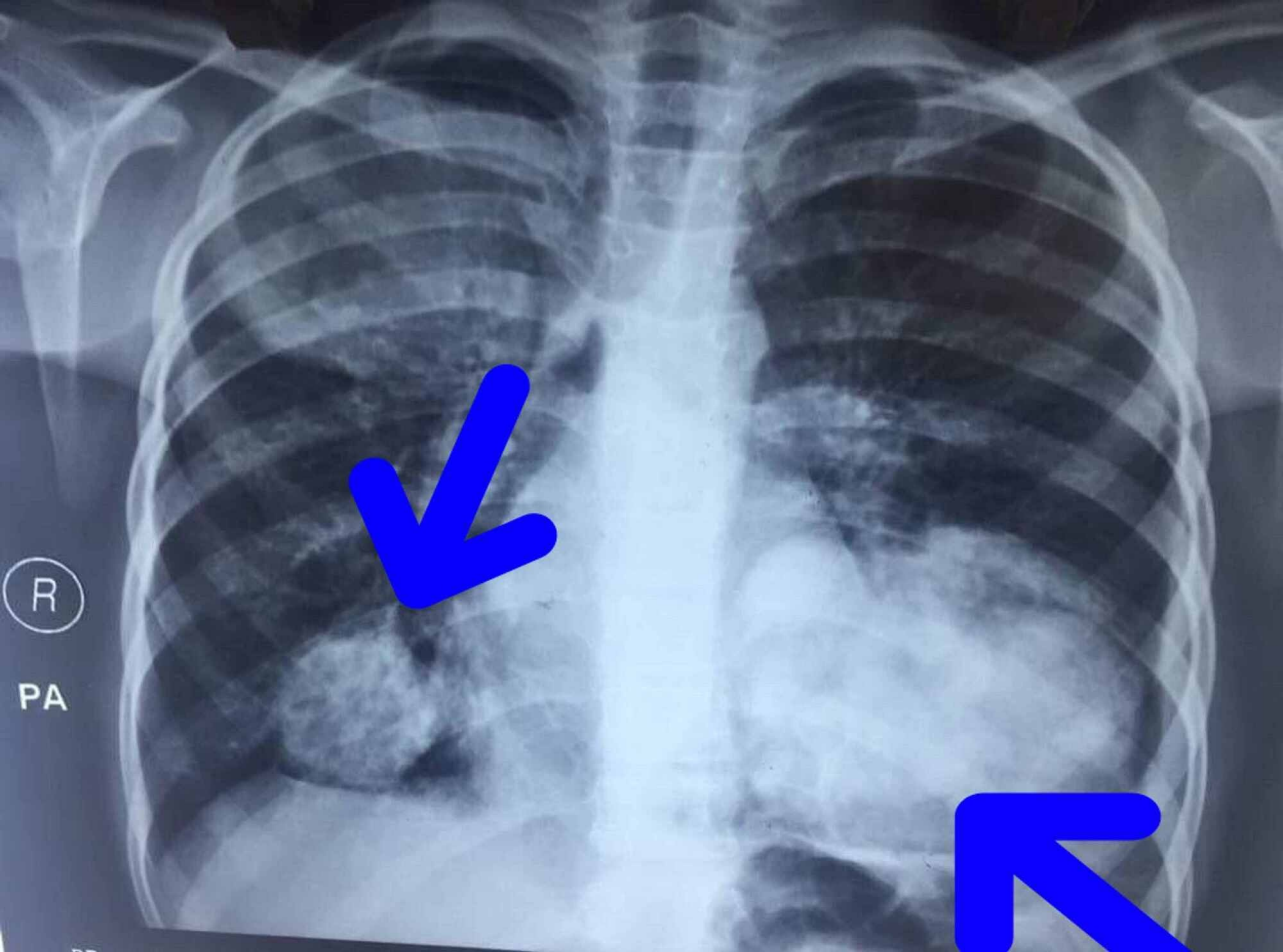
A chest X-ray revealed bilateral multiple dense masses of varying sizes and a small left-sided pleural effusion suggestive of metastasis

Given the findings in Figure 1 of multiple metastatic masses in the lung, an echocardiogram was performed to know the functional status of the heart and to rule out any congenital abnormalities.

**Figure 2:**
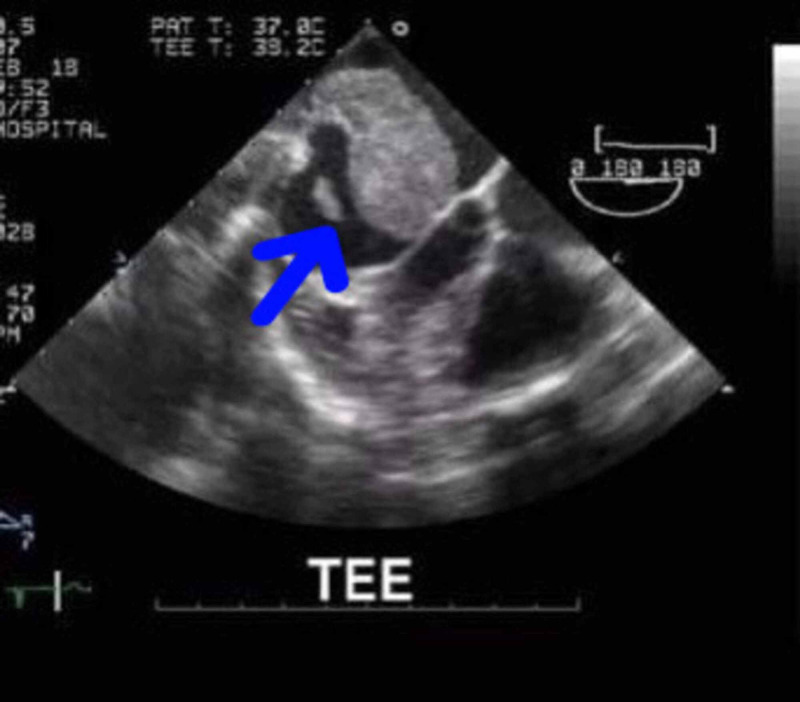
TEE before surgery TEE, transesophageal echocardiogram

**Figure 3:**
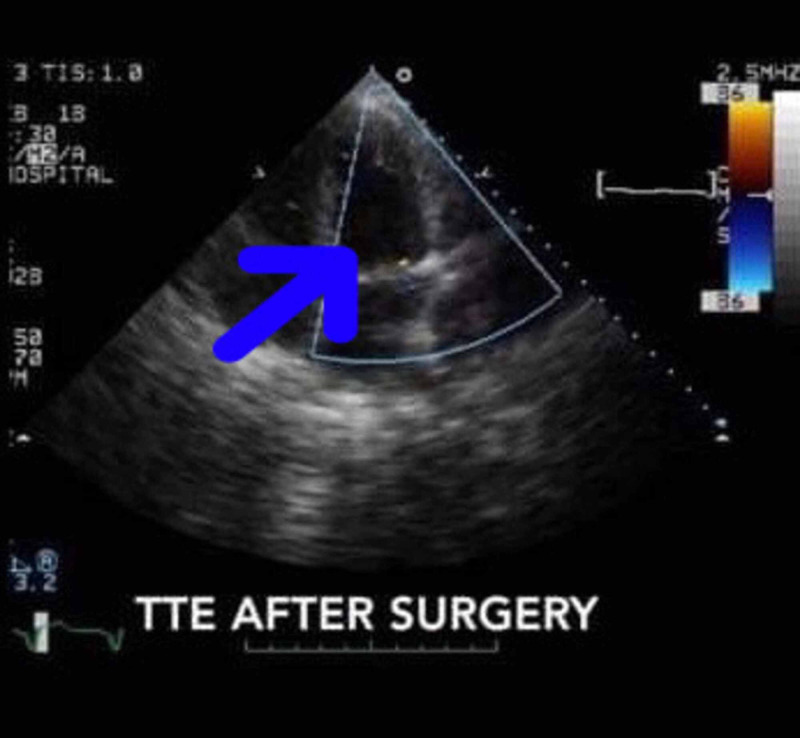
TTE after surgery TTE, transthoracic echocardiogram

Transesophageal echocardiogram and transthoracic echocardiogram depicted a dilated left atrium with a large mobile mass (1.62 cm by 2.45 cm) attached to the lateral free wall of the left atrium, suspicious for a neoplastic process. (Figures 2, 3). The mass was prolapsing across the mitral valve orifice in diastole, causing a functional mitral stenosis (mean diastolic transmitral gradient: 11 mm Hg) and mild-to-moderate mitral regurgitation. The right atrium and right ventricle were normal with mild tricuspid regurgitation (systolic pulmonary artery pressure [SPAP]: 22 mm Hg). A mild pericardial effusion was also seen with preserved left ventricular function and no wall motion abnormalities.

The cardiology team was consulted to assess the neoplastic lesion and based on the chest X-ray and echocardiography findings, the mass was suggestive of left atrial myxoma. Cardiovascular surgery was consulted for the resection of the left atrial myxoma. The patient underwent cardiac surgery five days after the initial diagnosis for mass removal. Operative findings revealed that the mass was found to be involving the lower left pulmonary vein and extending inside it. Histopathological report of surgical specimen describes the mass as reddish rubbery tissue measuring 45 mm in greatest diameter with small round blue cell tumor with occasional rosettes, rejecting the hypothesis of a cardiac myxoma and instead suggesting a diagnosis of “neuroblastoma.” Chest, abdominal, and pelvis CT scans did not detect any other mass or lesion. Since extensive imaging failed to show any primary site, a diagnosis of metastatic neuroblastoma with an unknown primary site was concluded.

## Discussion

Neuroblastoma is undifferentiated tumors that arise from the neural crest cells designated for the development of postganglionic sympathetic neurons. It is the most prevalent malignancy among extracranial solid childhood tumors. Based on its embryological origin and anatomical locations, it is usually found in the adrenal gland and around sympathetic chains. While the majority of them are located in the abdomen, 50% of them originate from the medulla of the adrenal gland, and the rest may emerge in the thorax (20%), neck (5%), or pelvic region (5%). Also, approximately 1% of cases have an unknown primary site [3,4].

The median age at diagnosis is around 17.3 months; however, cases have been reported in the pediatric population of older age [1,2]. Prognosis depends on factors such as stage, age at presentation, histologic features, and molecular and cytogenetic alterations such as N-Myc amplification of tumor. By comparison, young children have a good prognosis and even spontaneous regression without any treatment, whereas neuroblastoma in older children appears to have wide clinical manifestations, a more aggressive disease with metastatic potential, and an unfavorable prognosis regardless of the stage [1].

The initial presentation in young children includes high temperature (25%), abdominal pain (22%), abdominal mass (19%), and bone pain (19%) [9]. By contrast, neuroblastoma in adolescents is indolent, where half (50%) of the patients present with metastatic disease, and signs and symptoms vary according to the location of the tumor. There have been a few reports of neuroblastoma in the older age groups who already have had metastatic dissemination to unfamiliar sites and were later on diagnosed with a tumor based on histopathological evidence. This explains the case of our patient who presented with a mass in the left atrium, and microscopic evaluation of the excised mass revealed it as neuroblastoma.

Primary cardiac tumors are unusual, with an incidence rate of less than 0.1%. The most common primary neoplasm is myxoma in adults, whereas rhabdomyomas occur frequently in children. Myxoma has a predilection for the left atrium, and the initial presentation includes non-specific and variable symptoms such as fever, weight loss, and anorexia. However, secondary tumors are more common than primary tumors, and metastatic dissemination to the heart is still rare. In the presented case, a 12-year-old male child, who presented with constitutional symptoms as the initial presentation with a diagnostic challenge, was misdiagnosed with left atrial myxoma. It is difficult to differentiate this case clinically from other cardiac tumors, but histological findings of small, round, blue cells with occasional rosettes confirmed the diagnosis. Once the diagnosis had been made as neuroblastoma, the next challenge was to determine the origin of the occult tumor using imaging modalities and immunohistochemistry. Our patient’s imaging studies, which included CT, US of the abdomen, and bone scan, did not detect a measurable primary tumor. However, a chest X-ray revealed multiple lesions in the lungs, suggesting the possibility of seeding to the left atrium through a hematogenous route.

Regardless, cardiac neuroblastoma is extremely rare and is usually a result of the intracavitary cardiac extension of neuroblastoma, making it vulnerable to be missed [8]. Until now, in the literature, there is one case report by Bauchinger et al. that explains the spread of neuroblastoma (primary in renal) to the right atrium through tumor cells inoculating inferior vena cava [8]. In the cardiac mass found in our patient, with the appearance of a tumor based on echocardiography, mobile mass in the left atrium invading the left pulmonary vein first raised the possibility of atrial myxoma. The patient was proceeded with the treatment of surgical resection to prevent future embolization as indicated. However, the management of neuroblastoma is based on the staging system and requires a multimodal approach.

According to the International Neuroblastoma Staging System (INSS), cardiac involvement is considered a stage 4 disease [10]. Management of neuroblastoma depends on staging based on risk stratification, imaging, and immunohistochemical investigations. Surgical resection is considered in low-risk disease. For a high-risk patient, as in our case, surgery along with chemotherapy, radiotherapy, stem cell transplantation, and immunotherapy are recommended [11,12].

## Conclusions

As of now, in the literature, there are very limited data regarding metastasis of neuroblastoma to the left atrium with no known primary site, and there is a high probability that it can be neglected as a potential metastatic site by physicians. Analyzing the unusual spread will assist oncologists in understanding the disparities in the clinical behavior of neuroblastoma in older children. A multidisciplinary team of pediatric oncologists, surgeons, and radiation oncologists is important for the most effective outcome for patients.
